# Are the view of *Helicobacter pylori* colonized in the oral cavity an illusion?

**DOI:** 10.1038/emm.2017.225

**Published:** 2017-11-24

**Authors:** J K C Yee

**Affiliations:** 1 Research Lab of Oral H pylori, Everett, WA, USA

## Abstract

Urea breath test (UBT), as a leading preferred non-invasive diagnostic technology, but may not be able to detect oral *H. pylori*. With negative results of UBT, the patient may have an oral infection. On the basis of the fact of success, eradication rate may increase by 21% in the 95% Cl range after the elimination of oral *H. pylori, the* author believes oral *H. pylori* does exist and the oral cavity is the second colonized site aside its primary site of the stomach. *H. pylori* migrated out of Africa along with its human host *circa* 60 000 years ago; they are not lives in stomach only. In this review article, evidence established in recent years studies with use more appropriate technology had been listed and discussed. The author considers the oral cavity is a black hole for *H. pylori* infection that significant effective on gastroenterology and another medical field. The role of the oral cavity as the source of *H. pylori* infection is so controvert in past years. It seems like a human being having a second-time face to discover *H. pylori* in the history.

## Introduction

Most scientists in this field proposed there are no living *H. pylori* lives in the mouth and positive response of the oral cavity by PCR due to reflux from the stomach. The survive of *H. pylori* in the oral cavity lives for only a few hours. If this proposal is right, then the dead bacteria should not have any negative effective on eradication of stomach *H. pylori* infections. However, there are a number of reports that indicated eliminated oral *H. pylori* may help more patients recover from stomach infection.^1, 2^ These reports concluded that there is a significant relationship between stomach and oral *H*. *pylori* infection. This finding greatly explains why the annual *H. pylori* recurrence rates were so high with the first year being 13.2% followed by the second-year and the third-year both being 18.4%, due to oral cavity infection.^3^ In developed countries, the *H. pylori* recurrence rates after successful eradication were very low—which means that discrepancy exists and is likely determined by economic status. However, it still continues to be a considerable controversy on the fundamental issue of oral *H. pylori*. For example, how the stomach infection occurs? *H. pylori* stay in the mouth, then via the oral cavity come down to stomach? What is the premium function of the oral cavity in this process? Or the mouth is a second colonization site of *H. pylori*? Hence, if the mode of *H. pylori* transmission remains unknown, we are unable to interrupt the spread of infection. The resolution on these disputable issues is so important because the majority of physicians and scientists in this field do not consider oral *H. pylori* are living bacteria. I estimate ~20% of the population of Asia having *H. pylori* of oral cavity.^4^ There are ~280 million people of China having oral *H*. *pylori* infection. There are 280 million people have the problem of recurrences of stomach *H. pylori* infection. The abuse and overuse of antibiotics occur everywhere.^1, 3, 4^ As today, antibiotic pollution that appears in food, water, even in children’s urine that become a serious concern.^5^ If we do not stop this abuse, antibiotic may kill 80 000 per year and cost 11.7 billion dollars as medical expenses in China. This review will provide facts that indicating oral *H. pylori* infection exist and discuss how to eliminating them without antibiotic.

## The close relationship between periodontal health and *H*. *pylori* infection

Dye BA *et al.*^6^ report that a clinical periodontal study on 4504 participants during 10 years period that show the close relation between the depths of advanced pockets and a positive blood test for *H. pylori* antibodies. They concluded that poor periodontal health having periodontal pockets >5 mm always associated with *H. pylori* infection of adults in US population.^6^ Fernández-Tilapa G *et al.*^7^ use blood *H. pylori* antibody tests found that the prevalence of *H. pylori* in oral cavity was higher (18.5%) among seropositive subjects compared with seronegative persons in México. However, they concluded there were no association between the presence of *H. pylori* and oral hygiene habits. Furthermore, Nisha KJ *et al.*^8^ reported that *H. pylori* may colonize in dental plaque that shows a strong relation with periodontal diseases. Tsami A *et al.*^9^ also detected *H. pylori* presenting in the subgingival dental plaque of children as well as their family.

Several reports have indicated that *H. pylori* colonies could be grown only from root canals but not from plaque. The root canals of endodontic-infected teeth could be a reservoir for living *H. pylori* that could serve as a potential source of transmission.^10, 11^

Recent studies have shown that they consistently found *H. pylori* live in the oral cavity that strong like with recurrence of stomach *H. pylori* infection. Furthermore, in a review article, they indicted the treatment of periodontal disease may help the improvement of the symptoms of *H. pylori*-positive dyspeptic patients.^2^ A large size of a clinical study of China shown a high percentage of adults suffers from oral *H. pylori* infection, which also strong like with oral diseases; such as periodontal diseases and caries.^12^

A review and Meta-analysis included 48 articles and 12 clinical trials, as well as a meta-analysis designed in 2011 that indicated a strong association between *H. pylori* infection of mouth and *H. pylori* stomach infection. They found *H. pylori* are the etiologic agent of periodontal disease. They concluded that there is a strong relation between mouth and stomach *H. pylori* infection.^13^ Recent several meta-analyses report a similar conclusion regarding oral cavity and stomach.^14, 15, 16, 17^ The total participants involved in the above meta-analyses are more than 20 000 individuals (Table 1).

Furthermore, combining full-mouth disinfection plus triple therapy on periodontitis patients for oral *H. Pylori* infection increasing eradication success rate of stomach *H. pylori* infection.^18^

Although meta-analysis provided a right direction of periodontal diseases and *H. pylori* infection, however we should also discussion each individual study further since they reported a correlation in result but with negative conclusion, For example, A clinical study shows *H pylori* was detected in specimens of 34 patients (54%). Because all of the cultures of dental plaque were negative. They concluded that dental plaque or dentures are not an important reservoir for *H pylori* and are probably not a significant factor in transmission of the organism.^19^ Author did not agree with their conclusion because the method of cell cultures used for high concentration of *H. pylori* in stomach not for oral cavity where the concentration of *H. pylori* at very low level. Author will discussion why failure of cell cultures occur in later section.

However, there are a number of published articles that indicate there were no correlations between *H. pylori* gastritis and dental hygiene or periodontal disease. They concluded that either dental plaque or dentures have nothing to do with stomach *H. pylori* infection. At least, they consider oral *H. pylori* is not a significant factor cost stomach infection and they areorganisms in transmission in the mouth. However, the key fact is that they did indeed detect *H. pylori* in the oral cavity, but with a different view of conclusion.^20, 21^ They think the results of such studies should be considered prudently because the oral cavity is the residence of several urease-producing species, including S*treptococcus* spp., *Haemophilus* spp. and *Actinomyces* spp. Those organisms also have high urease activity in dental plaque which is nothing to do with oral *H. pylori*. While the diagnosis of *H. pylori* in gastric samples you may see the microscopic appearance such as Gram-negative, curved or spiral-shaped rods which may not as *H. pylori*. If you use microscopic appearance as a standard to check oral samples you can found many species have spirochetes appearance, including *Treponema* spp. so this standard has low specificity. We should be prudent to make any conclusion.^22^

Namiot *et al.*^23^ conducted a clinical study. They reported in 65.6% of an adult having *H. pylori* antigens exist in dental plaque. They concluded the occurrence of *H. pylori* antigens of dental plaque of natural teeth is not link with oral health status. After remove dental plaque of natural teeth and removable dentures, the H. pylori still stay in mouth. However, Silva *et al.*^24^ reported *H. pylori* was existing in the supragingival plaque, but not in the subgingival plaque in the case if the patient has periodontal disease and upper gastric diseases. They concluded *H. pylori* may colonize in the supragingival site and it is strong like with oral hygiene. Chaudhry *et al.*^25^ suggested use two genes of the bacterium simultaneously amplified as compared to one gene amplification only then we have better chance to found *H. pylori* in dental plaque which may be a reason why some report found no *H. pylori* in dental plaque.

Yang J *et al.*,^26^ reported a clinical study including 212 Han Chinese non-smoking adults. The results indicated that *H. pylori* positive status significantly increased the risk of periodontal diseases. Following is a summary table that listed all articles published from 1995 to April 2016 that indicated a close association of oral *H. pylori* and periodontal diseases in various countries with a data of a total of 61 299 individuals (Table 2).

In recurrent aphthous stomatitis, there was a strong relationship between oral *H. pylori* infection and stomach infection. Also, *H. pylori* may play an etiological role.^27^ An article indicated oral *H. pylori* may be associated with leukoplakia and lichen planus oral lesions.^28^

Since oral *H. pylori* infection has associated with stomach diseases, they reported periodontal treatment may have positive effective on systemic drug therapy that increasing eradication success rate on stomach treatment and reducing recurrence of stomach *H. pylori* infection.^29^ They found the *H. pylori*-positive rate in the healthy periodontal group was 15.38%, but it was 72.73% in periodontitis group was. They consider the dental plaque can be one of the main causes of recurrence infection of stomach *H. pylori* infection. The oral *H. pylori* can also be the source of oral–oral transmission.^30^ Conclusively, a new strategy which concomitant eradication in oral and gastric infection can result in clearance of *H. pylori* infection.^31, 32^ Further reports found same strain of *H. pylori* simultaneously exists in plaque and gastric mucosa. There was a positive correlation between the collected indices and quantity of *H. pylori* colonization.^33^ There might be a relation between oral of *H. pylori* and oral lesions. Therefore, they suggested that we should have an early detection and eradication of oral *H. pylori,* especially important in high-risk patients.^34, 35, 36, 37^ Cellini *et al.* found *H. pylori* exist in the esophagus and human saliva sample. They suggest that saliva and the esophagus may be a source of stomach *H. pylori* infection.^38^ Furthermore, Bago *et al.*,^39^ reported that almost half of the patients suffer from gastric *H. pylori* harbored the same bacterium in the oral cavity. After the eradication therapy of stomach infection, *H. pylori* was not detected in the oral cavity, they proposed high effectiveness of the therapy protocol in the oral cavity may not help stomach recover. They consider oral *H. pylori* as a transient character. However, Bago *et al.*^39^ is only one study that shows oral *H. pylori* was clean after eradication on the stomach, which did not agree with all remaining studies on this subject in past 20 years.

## The association between stomach and oral *H. pylori* infection

Whether the oral *H. pylori* are transient or permanent in the mouth, the fundamental question is, ‘Can oral *H. pylori* be a reservoir for gastric *H. pylori* infection?’ One might be first address whether there is an association between oral and gastric *H. pylori* carriage. Several studies have reported there is positive link oral with gastric *H. pylori*.^40, 41, 42^ Conversely, there was a study that indicated there were no such association.^43^ Song *et al.*^44^ reported that *H. pylori* exist in the oral cavity of 97% of patients that has characteristic distribution independent of the status of stomach infection. This is why they consider *H. pylori* may belong to the normal oral microflora, which is nothing link with stomach infection. However, recent studies show the bacterial involving oral cavity and stomach has an identical or closely species which related strains of *H. pylori* that provided a good evidence of the role of the oral cavity link with gastric infection.

One of the articles has a view against oral *H. pylori* link with stomach infection because they found oral and stomach *H. pylori* have different genotypes. This study shows that more than one *H. pylori* strain exists in the oropharynx and stomach at the same patient. They concluded that oropharyngeal infection is independent of the gastric infection.^10^ However, remarkable genotype diversity among stomach, saliva and stool that showed that more than one *H. pylori* genotype may exist in the same patient.^45^ However, there is an article reported the same strain of *H. pylori* simultaneously exists in plaque and gastric mucosa.^34^

There is increasing evidence recently regarding the role of the oral cavity in the transmission of *H. pylori to stomach* use new methods to detecting *H. pylori* in the oral cavity. That evidence continually supporting the view of the association between oral and stomach *H. pylori* infection. But, Young *et al.*^46^ Consider this subject requires considerably more clinical studies before make a definite conclusion; especially we need a technology to confirmed oral *H. pylori* exists. As long as we can confirm oral cavity involving, then we can do preventive measures oral transmission. Rasmussen *et al.*,^47^ report a strong link between oral *H. pylori* and gastric infection in Brazilian community. In their finding, the *H. pylori* exist in the oral cavity with different distribution between saliva and dental plaques that may suggest a potential link between oral infection and recurrence of stomach infection. Morales–Espinosa *et al.*^48^ report that in Mexico, many patients have *H. pylori* in the oral cavity suffer from gastric symptoms. Therefore, they suggest we should have the combination of treatments on both sites immediately.

There are several reports indicated eliminating dental plaque can significantly improving *H. pylori* of the gastric mucosa.^41^ Zaric S *et al.*^49^, report a combination periodontal treatment and drug systemic therapy can increasing the eradication success rate of stomach *H. pylori* infection and decreasing the risk of recurrence of stomach infection. Therefore they suggested that we should do professional plaque removal and oral hygiene procedures along with the antibiotic treatment of stomach *H. pylori*. infection.^50, 51^ Since the *cagA* gene exists in both gastric biopsies and saliva, as well as dental plaque^52^ this evidence further supports the view of a close association relationship between oral and stomach *H. pylori* infection. Al Asqah *et al.*^33^ conducted a clinical study that shows 65% of patients have dental plaque *H. pylori*. Among them, there were >50% patients’ harbored same bacteria in their stomach. In the same manner, the periodontitis patients had a significantly higher percentage of *H. pylori* in their dental plaque and the stomach that compared with patients without periodontitis. In addition, 78% of patients have *H. pylori* dental plaque in periodontitis group versus only 30% in non-periodontitis group. The coexistence of *H. pylori* in both dental plaque and the stomach had been observed.^53^ Liu Y *et al.*^54^ found dyspeptic patients with gastric infection are more likely to harbor *H. pylori* in their mouth that show a close association between *H. pylori* in the oral cavity and the stomach. Loster *et al.*^55^ found an interesting relationship of the lengths of dentist occupations. The dentist may carry gingival sulcus infection with *H. pylori* after a long time working on a patient with oral *H. pylori* infection that indicating oral *H. pylori* can be contagious through dental instruments. Yee *et al.*^56, 57^ had conducted several large clinical trials in China where there were >10 000 individuals involved. More authors in addition to Yee *et al.*^56, 57^ conducted similar clinical trial in various countries. All of them found a strong association of oral and stomach *H. pylori* infection Table 3.

However, Silva *et al.*^58^ had different viewpoints. Because they cannot found *H. pylori* in any oral samples who suffer from stomach *H. pylori* infection. Also, they found no genotype cagA in oral samples and cannot characterize vacA genotype in an oral sample of >30 patients.

## Why the traditional drug eradication of gastric *H. pylori* infection is ineffective against oral *H. pylori* infection?

In 1999, Dore-Davin *et al.*^59^ first discovered after systematical eradication on stomach *H. pylori* infection that had no effect on oral *H. pylori*. Miyabayashi *et al.*^60^ further reported the eradication success rate of stomach *H. pylori* infection was significantly lower in the oral *H. pylori*-positive cases compared with oral *H. pylori*-negative cases at 4 weeks after drug treatment. Two years later, they found 95.8% of patient had no stomach *H. pylori* infection with oral *H. pylori*-negative cases but they found only 69.5% of patient with no stomach *H. pylori* infection with oral *H. pylori*-positive. They concluded that oral *H. pylori* infection affected eradication successes rate and oral *H. pylori* infection has a strong link with a recurrence of gastric infection.^60^ Since 1999, there are numerous studies that show when patients received drug treatment on stomach *H. pylori* infection that cannot clean up oral *H. pylor*.^1, 32, 39, 61, 62, 63, 64^ All later reports support Dore-Davin’s first discovery (Table 4).

In terms of how to treat an oral *H. pylori* infection, there are a number of studies showing that mouth rinse treatment alone or combined with periodontal therapy may eliminate oral *H. pylori* infection and increase the eradication success rate of stomach *H. pylori* infection.^1, 2, 63^ A clinical study showed that the symptoms of *H. pylori*-positive dyspeptic patients may improve by oral treatmet.^2^ There are three studies^65, 66, 67^ that evaluated the effectiveness of periodontal treatment on *H. pylori* of mouth. They reported that patient received periodontal treatment may decrease plaque *H. pylori.* After treatment, if those patients still suffer from plaque *H. pylori* then followed a combination of treatment with triple therapy. Jia *et al.*^67^ proposal a periodontal treatment before eradication on stomach *H. pylori* infection. They reported that the prior periodontal intervention significant increasing eradication of stomach *H. pylori* infection of dyspeptic patients. After half year, the stomach *H. pylori* infection of the group received periodontal treatment has much lower than the group received no periodontal treatment. However, our studies^1^ shown that patients who received teeth cleaning had no effectiveness on *H. pylori* infection of mouth by statistical analysis. The special mouth resin is best effective in eliminating oral *H. pylori* infection that I will discussion it at the end.

The reason why eradication on stomach *H. pylori* infection has no effective on *H. pylori* infection of the mouth, because *H. pylori* exist in between the teeth and gums called, an area referred to as the ‘bio- film membrane’ (Biofilm), also we called as plaque barrier. The drug cannot penetrate it when the patient received symmetrically eradication. This is why the conventional treatment on stomach *H. pylori* infection had no effect on oral *H. pylori*; especially it exists in dental plaque.

## The eradication of stomach *H. pylori* infection faces more challenging than ever due to progressive loss efficacy of traditional therapy

There were several proposals how to providing treatment after failure of second-line therapies. One of them is the endoscopic-guided antibiotic susceptibility testing. However, according to the principal of Maastricht Guidelines, its role has expanded over, over again in past years. Several authors have reported the results of such proposal. The developed both efficacy clinical trials and cost-effectiveness trials against drug-resistant of treatment on stomach *H. pylori* infections. However, their results are not very successful, because antibiotic resistance is not the only main reason for the failure so far. The failure becomes a good attention in medical societies worldwide, special in Asia.^68^ It is time now we should establish a new view besides antibiotic resistance, which are the most important issues for the progressive loss of efficacy of eradication due to oral facts. Yee^69^ proposed the key conception that *H. pylori* have a second colonized site in an oral cavity in addition to the stomach. However, some authors said ‘oral *H. pylori* cannot be cultured’, ‘*H. pylori* exists in the oral cavity are dead bacterial that has no effect on stomach treatment’ and ‘the oral cavity is not a colonized site,’ which has become the main reasons to deny our version of oral *H. pylori* colonization. Because the majority of physicians working in gastroenterology field ignore the oral *H. pylori*, ~20% of the population of Asia suffers from oral *H. pylori* infection^1, 3^. Not only in Asia, Jonaitis *et al.*^70^ reported that after *H. pylori* eradication they frequency observe on *H. pylori* recurrence of peptic ulcer patients during 9 years in Lithuania The recurrence rate of *H. pylori* is high at 27.2%. This number is very close that we found in Asia.

**Figure 1:**
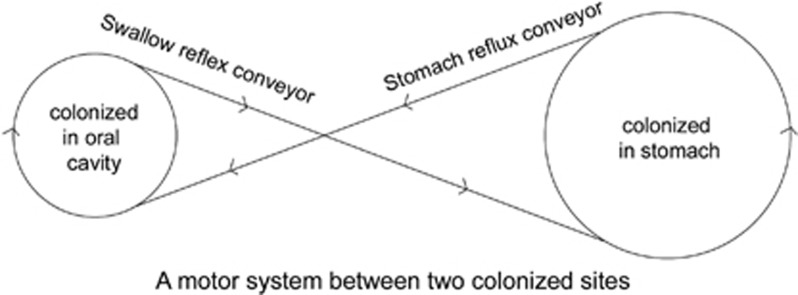
A motor system between two colonized sites.

There is a motor circulatory system that I summarized that behind the negative impact of oral *H. pylori on* stomach treatment (Figure 1). This system contains two colonized sites of *H. pylori* in the upper digestive system. The primarily colonized site resides in the stomach and delivers *H. pylori* into the oral cavity by reflux as a conveyor. The oral cavity is a second colonized site for the culture of *H. pylori;* bacteria from this site drop into the stomach by the swallow reflex as a conveyor. Two colonized sites with two conveyors have been constructed to create a system that transports *H. pylori* along the upper digestive system. One of the colonized sites contains *H. pylori*, which results in a second colonized site being occupied by *H. pylori*. The recurrence of *H. pylori* infection occurs if one site had been treated by a drug and another site had not. The motor circulating system can transport *H. pylori* along the circle. A number of studies have shown that oral *H. pylori* were not eliminated in patients who received a drug treatment for stomach *H. pylori*.^13, 63, 71^ Traditional drug eradication and teeth cleaning had an effectiveness rate of less than 10%. By statistical analysis, there was no effect all on oral infection. A new strategy that concomitantly eradicates oral and gastric colonization would result in clearance of *H. pylori* infection and improve the eradication rate of gastric *H. pylori*.^1^

The discovery of oral *H*. *pylori* is especially significant and meaningful because this motor system can explain why the recurrence of stomach *H. pylori* infection occurs.

## Urea breath test, a gold standard diagnosis, use only for diagnosis of stomach *H. pylori* infection

Urea breath test (UBT) C^13^ is a trusted diagnostic procedure used to identify stomach infections by *H. pylori*^72^ with the exception of a small number of false positives^73^ and they diagnosis for all *H. pylori* species not specific for CagA.^74^ The principle is based upon *H. pylori* to transform urea that released by *H. pylori* to carbon dioxide and ammonia. UBT is a popular method for diagnosis of *H. pylori* of the stomach. It holds efficacy at 96.7% sensitivity and 96.2% specificity. However, UBT is not used for diagnosis of oral *H. pylori* because C^13^ or C^14^ are not dissolved in the mouth during the testing. In medical practice, doctor considers you have no stomach *H. pylori* infection if you have negative results of UBT C^13^. In fact,doctor only pays attention to stomach infection. In their view, there is no *H. pylori* infection exist in anywhere besides stomach. This is traditional view on *H. pylori* infection for many years. However, the clinical study provides evidence that shown *H. pylori* oral infection are nonetheless present that also negatively effective on eradication on stomach *H. pylori* infection. In Asia, approximately 20–30% of the population having oral *H. pylori* infection but with negative UBT results.^1^ We developed a technology, *H. pylori* saliva test (HPS) that especially detecting oral *H. pylori*^4^ and it is non-invasive, fast result and no equipment required during the testing.

It is controversial the fact of *H. pylori exists* in the oral cavity in past 20 years. It divided scientists into two groups. The majority scientism belongs to the first group that proposed that *H. pylori* stay in the oral cavity and that all positive results detected by PCR are fragments of dead bacteria that reflux from the stomach which could not be cultivated from PCR-positive samples.^75^ The proposal says the oral *H. pylori* come from stomach reflux was survive only a few hours in the mouth. Because oral cavity holds high oxygen concentration that kills all *H. pylori.* If the proposed view is correct, then the fragmented of bacteria have no negative effect on eradication of stomach *H. pylori* infections.^75, 76^ However, their view contradicts with the studies of PCR recently published,^77, 78^ the fact of the oral cavity may have hypoxia environment,^35, 79^ the fact of oral *H. pylori* infection cannot clean up by traditional therapy.^32, 46^ The fact that indicated *H. pylori* can be cultured from saliva sample if we use a new method of culture.^1^ The fact of oral and stomach has the same gene of *H. pylori.*^7, 55^ It also contradicts with the evidence of the lower rate of eradication of stomach *H. pylori* when a person suffers from oral *H. pylori* infection^41^ and the fact of results of all meta-analysis published in the past.^13, 71^ Therefore, we proposed a new view that indicated *H. pylori* colonization of the oral cavity which may resolve all previous issues in past.

## What is the best test for detecting *H. pylori* of the oral cavity in clinical settings?

PCR is a method often used for detecting oral *H. pylori*, but its results have high variation. Some articles report the detecting rate were zero, but some articles report with 90% of positive response on the oral sample. Naturally, the scientific community very confused on the large variation of PCR testing results. We should find out why such discrepancies exist.^22^ In term of the requirement of the sample, expensive equipment required and technician for operating PCR testing. So PCR method is not a good and convenient way to detecting oral *H. pylori* for clinical settings. Therefore, a diagnostic method has a high sensitivity and specificity for oral sample should be established. We believe that HPS is a good and convenient test for diagnosis of *H. pylori* in the oral cavity. As long as we have an easy, accrue test, then the clinical trial can be carried out on a large number of patients to obtain a good size of clinical data, which will help to understand the strong links between oral and stomach *H. pylori* infection. We are able to establish the principles of evidence regarding oral *H. pylori* infection.

HPS is a lateral flow immunochromatographic test device that uses saliva as testing sample detecting oral *H. pylori* within few minutes. The principle of HPS is based on monoclonal antibody react with oral urease produced by *H. pylori*.

A laboratory study was conducted to determine its specificity. The following common bacteria of oral cavity were applied: *Streptococcus gordonii*, *S. mutans* (major pathogen of dental caries), *S. salivarius*, *S. sanguinis* and *Veillonella parvula, Porphyromonas gingivalis* (major pathogen of periodontitis), *Gemella haemolysans*, *Granulicatella adiacens, Campylobacter rectus* (major pathogen of periodontitis, species related to *Helicobacter*), *Corynebacterium matruchotii*, *Bifidobacterium dentium, Actinomyces naeslundii*, *A. odontolyticus.* All the above bacteria did not show interference or cross-reactivity with HPS test.

The sensitivity of the tests was 10 ng ml^−1^ *H. pylori* antigen.^4^

## Do oral *H. pylori* come from stomach reflux?

Do *H. pylori* in oral cavity come from stomach? That is one of the key issues that had been disputed in past. The author had a discussion on this question with the Nobel Laureate, Dr. Robin Warren. He indicated that oral *H. pylori* have to have come from the stomach through the reflux motion. In the case, if the patient had no *H. pylori* of the stomach, then why oral *H. pylori* exist? He believes very low numbers of bacteria lives in the stomach, but show false-negative UBT tests. They can through reflux the bacteria back to the oral cavity. Since scientist cannot culture on a positive sample by PCR that leading him to be trusted there are no living *H. pylori* in the oral cavity. His past lab experience also shows that he never managed to culture *H. pylori* from food, water or the mouth. He also advice when we say antigens of saliva or plaque, we should very carefully tell what methodology that to demonstrate those antigens. Another word he did not trust our new technology, HPS can detect oral *H. pylori.*

If a patient with *H. pylori* infection of the stomach, then it is a good reason to believe the oral bacterial come from stomach reflux. But, with a UBT-negative patient and negative culture of stomach sample, we still detected *H. pylori* in the mouth. Also, we observe a large number of the patient which near 10 000 patients with negative UBT test in several clinical studies but see oral *H. pylori*. This is why we confidence our data are reliable.

If we confirmed *H. pylori-*colonized in the oral cavity by culture saliva sample? What will be our next step? To follow up, we have conducted several studies in several subjects (1) What is the recurrence rate of stomach *H. pylori* infection each year to oral *H. pylori*? (2) Is it true that systematically drug treatments are not effective on oral *H. pylori* due to the construction of dental plaque structure? And (3) is the eradication rate of stomach *H. pylori* infection getting lower each treatment?

## Culture of oral *H. pylori*

Krajden *et al.*^80^ in 1989 first reported the result of the culture of *H. pylori* gastritis. There was only one plaque had a positive result among seventy-one patients. All seventy-one saliva cultures show negative result. Since then, many attempted to cultivate oral *H. pylori* had been rarely successful worldwide (Table 5).^19, 30, 53, 81, 82, 83, 84, 85, 86, 87, 88, 89, 90, 91, 92, 93, 94, 95, 96, 97, 98, 99, 100, 101, 102, 103, 104, 105, 106, 107^ Indeed, published articles reported culture-positive rates are very low from various oral sample included saliva, dental plaque, and teeth. Majmudar *et al.*,^97^ D'Alessandro and Seri,^102^ reported that they had been successfully cultured on saliva; however, Namavar *et al.*^101^ consider their results were false positives. The main difficulties of bacteria culture of the oral sample; are how to collect oral specimen; how to preserve it; there was a small number of colonies of *H. pylori* for culture and competition with other oral bacteria. Because the concentration of *H. pylori* of the stomach is three magnitudes higher than that of the oral cavity (10^5^ CFU per ml versus10^2^ CFUper ml^108, 109, 110^), it would be insufficient to use conventional stomach culturing techniques for detecting oral *H. pylori*. The method must be adapted to obtain a high positive rate of oral *H. pylori* culture with very low concentration of the oral sample. However, Dowsett *et al.*^111^ dispute that If such low concentration of *H. pylori* in the oral then it will be an insufficient number of bacterial result infection in any where. If this is indeed the case, the subsequent ability of oral *H. pylori* has negative on stomach infection that may be questionable.

Author’s lab had been culture *H. pylori* on saliva successfully since 2012*. H. Pylori* were isolated from saliva by pretreatment with Urea-Hydrochloric acid.^108^ We performed all the following tests to confirm *H. pylori* colonization:(a) Oxidase test (b) Catalase test (c) *H. pylori* antigen and antibody test (d) Microscopy observations.

I then communicated with Dr. Floyd E. Dewhirst, a significant scientist related to this field^112^ regarding confirmation on the result of *H. pylori* culture. He indicated that **t**his is not sufficient; we should have a full 1500 base 16S rRNA sequence of the isolate(s), and deposit the organisms in a national culture collection. With sequences and strains, the work is much stronger. He suggested the existing published studies show *H. pylori* can be cultured^19, 30, 53, 81, 82, 83, 84, 85, 86, 87, 88, 89, 90, 91, 92, 93, 94, 95, 96, 97, 98, 99, 100, 101, 102, 103, 104, 105, 106, 107^ were not accountable because of none of them involved work with a full 1500 base 163r RNA sequence. He further indicated that the confirmation of culture results do not address transient (from burp) versus colonization. The oral microbiome and the gut microbiome each have greater than 700 species. The number of species shared between these two different habitats is one organism (Dialister pneumosintes). Even though vast quantities of oral bacteria are swallowed every day, and none (except one) colonizes the GI tract. Organic bacteria are very specific in their niche selection. There are nine niches in the oral cavity—sub-gingival, supra-gingival, tongue, tonsils, throat, attached gingiva, cheek, palate and saliva—and each niche has a distinct microbial (some overlap). The oral cavity has no site with gastric mucus and a pH of close to 1. Where do we think it colonizes? Dr Dewhirst examined 27 subjects at the nine oral niches and reviewed the site sample reads by illuminate 16S rRNA of the V1–V3 region. The region had about 100 000 reads per site, so 100 reads are 0.1% of the total, plus he never saw *H. pylori* in any subject, at any site. Maybe his subjects did not have *H. pylori* in their stomach—this was not the point of the study. However, if we believe it is part of the microbiome in the mouth, then we need to show where in the mouth and at what percent of the population. If we find it at a high concentration in some oral niche, then fine—we can say we see it in at least one person at that particular concentration in this site/niche. However, this still does not address the question of transient versus commensal (or indigenous microflora). However, the author considered the clinical trial of eliminating *H. pylori* of the oral cavity which increase the successful rate of eradication of stomach *H. pylori* infection, and the best evidence of *H. pylori* in the oral cavity is commensal microflora.^113^

## Are *H. pylori, a* sexually transmitted bacterium?

If the view and evidence of *H. pylori*-colonized in the oral cavity are correct, then oral *H. pylori* can be sexually transmitted bacteria. On the other hand, if no *H. pylori* sexually transmitted diseases through oral exist, how we can believe that *H. pylori* exist in the oral cavity? Let us check the reference here to see what they reveal it.

Use blood *H. pylori* test run a clinical study on sex partners with man and woman. The results show *H pylori,* the non-infected individual had statistically significant different prevalence rates of sex diseases. There were 83.3% vs 28.5%, respectively,^113, 114^ which may indicate *H pylori* may be a sex transmitted bacterium.

Several studies have shown there is a strong relationship between sexually transmitted disease and ethnicity minorities’ groups.^115, 116^ They report the high rates of sexually transmitted infection occur in ethnic minorities.

Schutze *et al.*^114^ reported the *H. pylori* infection are contingent and transmitted between spouses. They found a recurrence of *H. pylori* spouses contains same identified strain gene type from spouses which is a good evidence that indicated *H. pylori* transmitted among spouses. However, they also found multiple strains exist in the same individual.

There were very limited articles published regarding sexual transmission of *H. pylori* among female sex workers. Eslick GD^117^ reported that so far even there is no study conducted a prevalence of *H pylori* infection among female sex workers.

By theoretical analysis, they proposal *H. pylori* may colonize in the vaginal associated with yeast which constructed as biofilm formation, based on *H. pylori* exists in the biofilms among many bacterial species. This is why treatment failure occurs on yeast infection of the vaginal site. Eslick further hypothesized that *H. pylori* may colonize in an acidic vaginal environment that making vaginal as a source for sexual transmission for many species of bacteria.^118^

It was interesting an early case report that said they found ‘spiral bacteria’ with same strains of *H. pylori* in a woman’s vagina who suffers from vaginitis. They describe spiral bacteria have comma-shaped rods (1–4 μm in length) with a corkscrew motion and its head bear with four to eight flagellae. This finding was a year before original article of Warren and Marshall that discovered on stomach *H. pylori* infection. Besides their appearance was similar, they also found that some of those spirals bacteria can be cultured under microaerophilic condition after 72 h incubation at 37 °C. The biochemical profile was very similar between spiral bacterial and *H. pylori*, but they did not run a further test to confirmed both of them are same species. Several articles indicated vertical transmission of *H. pylori* exists in the vagina during the birth process. The prevalence of *H. pylori* in pregnant women is about 20%.^119, 120^

Kast RE reports a case that oral directly contacts with the nipple that may result in the retrograde propulsion of *H. pylori* into breast ducts which may lead to fibrocystic breast changes. It is a heterogeneous group of benign. In this case report, the woman had an *H. pylori* serology diagnosis as negative. However, after antibiotic eradication, she had no more pain and tenderness in breast and her breasts normalized.^121^ This was the reason leading his hypnosis that this woman had *H. pylori* retrograde into breast duct results *H. pylori* local infection. An article reported that mothers had been diagnosis with *H. pylori* antigenuria, the fecal of their half of breastfed 3 days old neonates found *H. pylori*.^122^ Are *H. pylori* transmitted from mother to neonates? Or from vertical transmission through vaginal delivery? They also found *H. pylori* exist in 4 out of 66 milk samples of mother suffered from *H. pylori* infection.^123^

Since oral sex is a very popular worldwide toady, the action of fellatio from woman to man, the *H. pylori* can transmit into the urethra that resulting infection. The question is why a large proportion of males suffers with non-gonococcal urethritis, but no responsible bacteria found? This was an article suggested we should link urethritis with *H. pylori* infection and urethritis.^124^

When I have a personal conversation with Dr Kast RE who hypnosis on the link between prostate and *H. pylori* infection because fellatio was so popular.

An article reported vaginal yeasts as primary reservoir of *H. pylori* that may facilitate transmitted it to neonates. Mother with UBT positive may transmit *H. pylori* through vaginal delivery to neonates based on there are close associated oral yeasts of neonates and vaginal yeasts.^125^

Healthcare workers special baby delivery workers are more careful on *H. pylori* infection because it is contagious during vaginal delivery procedures.

An article indicated *H. pylori* may transmit through fellatio in the urethra. However, they proposal further research is required to defined the link between in *H. pylori* and urethritis.^126^

## Prevention oral–oral transmission of *H. pylori*

Chow *et al.*,^127^ found a strong association between prevalence of *H. pylori* infection and chopsticks user in Chinese society of Australia. They consider the saliva containers with *H. pylori* through chopsticks.

On the basis of the fact of oral to oral transmission, water carry and fecal to oral transmission, Dowsett *et al.* indicated *H. pylori* infection have been population dependent.^128^ A report from India that indicated there was a significant association between *H. pylori* infection and fingernail carriage based on *H. pylori* had been detected by PCR on beneath of fingernails. It is a customer of eating way in India. They use a finger to hold food. So washing hands before eating may be important for stop *H. pylori*’s transmission.

By blood *H. pylori* antibody study, they found a strong link between *H. pylori* infection and crowded living condition.^128^ They further consider the socioeconomic status become an important issue that influences their finding. In developing countries, special in low socio-class children suffer from *H. pylori* infection become an important issue. There are no symptoms at all during a long period of latency until adult. Same way as stomach cancer resulted by stomach *H. pylori* infection usually does not show until older age.^129^ Regarding *H. pylori* transmission, mother as *H. pylori* carrier may be the main source for childhood *H. pylori* infection.^130^ A review article^131^ reported the prevalence rates vary widely with different ethnic groups and geographical location. An oral–oral route of transmission had been mentioned with all studies that indicated a fact of *H*. *pylori* exists in the oral cavity.

Dowsett and Kowolik^22^ dispute that if the fact of *H. pylori* transmission through oral to stomach, then we naturally expect oral *H. pylori* infection should be more often than stomach *H. pylori* infection. Our studies have shown the discovery of oral *H*. *pylori* is especially significant and meaningful that may answer the question raised by Dowsett and Kowolik, because it is ~20% of the population of Asia suffers from oral *H. pylori* infection.

## Non-antibiotic treatment for eliminating oral *H. pylori*

There is a non-antibiotic treatment for oral *H. pylori* infection available.^1^ Our studies indicated e-polylysine (L) and the Glycerol Monolaurate (GM) may eliminate oral *H. pylori.*

The L holds around 30 L-lysine residues. They use ‘e’ as a link with all Lysine molecules. Its surface has cationic. From electronically point view, the surface of *H. pylori* charges with positive power. The lysine amino acids are molecularly linked by the epsilon amino group and the carboxyl. If lysine meets with *H. pylori* in water, they will be having the very strong electronically huge power that making a cell of *H. pylori* attached with lysine molecules. The many liners of lysine molecules have clockwise and counter clockwise motion like a knife that tear membrane of *H. pylori* as fragments. The oral *H. pylori* will die.

**Figure 2:**
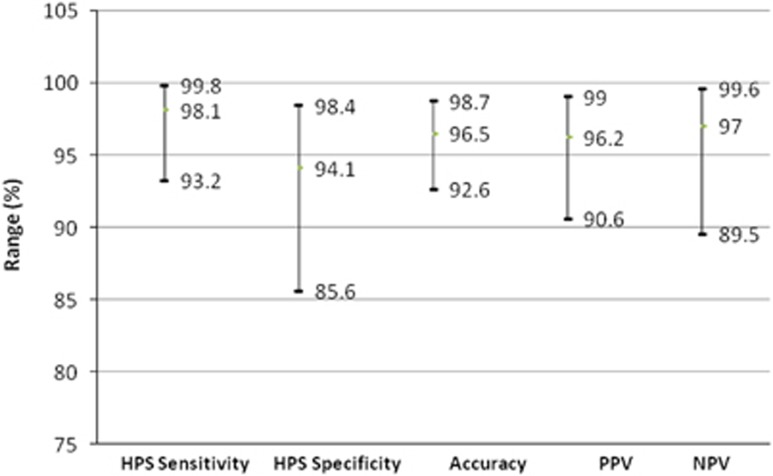
The accuracy of HPS confirmation by S-HP-C.

We found GM exist in mother’s milk that is nature preventive material for human's milk that formed by glycerol and lauric acid. In the water, GM will kill *H*. *pylori* immediately. We use Lysine and GM, called L-GM formed in mouth washing solution treat oral *H*. *pylori* infection. The patient uses it twice a day and 20 cc each time with 5 min in the mouth. After 2 months, we found increasing efficacy of eradication on stomach infection about 21%.^1^ These results of improvements had been confirmed by Saliva *H. pylori* culture (S-HP-C; Figure 2). On the basis of the confirmation of S-HP-C, we calculated the sensitivity, specificity, accuracy and positive and negative predictive values of HPS as 98.1, 94.1, 96.5, 96.2 and 97% respectively.

## Conclusion

A colonized site of *H. pylori* can exist in the oral cavity.

In medical practice, doctor consider patients had no stomach *H. pylori* infection. But in fact, patients can have negative results of UBT, but *H. pylori* exist in oral cavity. UBT cannot detect oral *H. pylori*.

If there is a live *H. pylori* colony in the oral cavity, then it would have a negative influence on the eradication of a stomach infection. In the classic *H. pylori* eradication programs, there are no clear measures of oral *H. pylori*; frequent relapses become more critical.

## Figures and Tables

**Table 1:** Meta-analysis of periodontal disease and oral *H. pylori* associated with stomach *H. pylori* infection published during 2011–2016

*Author*	*Year*	*Number*	*Conclusion*
Ren *et al.*^15^	2016	691 participants	Periodontal therapy increased eradication rate of stomach infection
Sayed *et al.*^16^	2014	4959 participants. Articles published during 1990–2012	Oral *H. pylori* increased stomach re-infection
Adler *et al.*^13^	2014	>5000 participants 48 articles and 12 clinical trials	Close relation oral *H. pylori* and stomach *H. pylori* infection
Marbaix *et al.*^14^	2013	> 5000 participants Included 48 articles	Close relation oral *H. pylori* and stomach *H. pylori* infection
Navabi *et al.*^17^	2011	1861 participants	Close relation oral *H. pylori* and stomach *H. pylori* infection
Zou and Li^71^	2011	>6000 participants Articles published during 2010–2011	Close relation oral *H. pylori* and stomach *H. pylori* infection

**Table 2:** All articles published from 1995 to 2016 regarding the discussion on periodontal disease associated with oral *H. pylori*

*Authors*	*Type*	*Country*	*No*	*Methods*	*Conclusion*
Nisha *et al.*^8^	Original Research	India	500	Seropositive	Periodontal close to *H. pylori*
Ren *et al.*^15^	Original Research	China	691	PCR	Periodontal close to *H. pylori*
Yang *et al.*^26^	Original Research	China	212	PCR	Periodontal close to *H. pylori*
Gulseren *et al.*^27^	Original Research	Turkey	81	RUT	Periodontal close to *H. pylori*
Kazanowska *et al.*^28^	Original Research	Poland	126	PCR	Periodontal close to *H. pylori*
Zheng and Zou^18^	Original Research	China	70	PCR	Periodontal close to *H. pylori*
Veiga *et al.*^20^	Original Research	Portugal	447	PCR	No close relation
Ding *et al.*^12^	Original Research	China	1050	HPS	Periodontal close to *H. pylori*
Ogaya *et al.*^11^	Original Research	Japan	40	PCR	Periodontal close to *H. pylori*
Lauritano *et al.*^29^	Original Research	Italy		PCR	Periodontal close to *H. pylori*
Amin *et al.*^31^	Original Research	Iran	45	PCR	Periodontal close to *H. pylori*
Anand *et al.*^46^	Review Article	India		PCR	Periodontal close to *H. pylori*
Adler *et al.*^13^	Review Article	Argentina		PCR	Periodontal close to *H. pylori*
Abadi *et al.*^32^	Original Research	Iran	134	PCR	Periodontal close to *H. pylori*
Yang *et al.*^33^	Review Article	China		PCR	Periodontal close to *H. pylori*
Bharath *et al.*^34^	Original Research	India	56	PCR	Periodontal close to *H. pylori*
Al Sayed *et al.*^16^	Review Article	India		PCR	Periodontal close to *H. pylori*
Boylan *et al.*^36^	Original Research	USA	49120	Period exam	Periodontal close to *H. pylori*
Marbaix S *et al.*^14^	Review Article	France		PCR	Periodontal close to *H. pylori*
Irani *et al.*^35^	Original Research	Iran	228	PCR	Periodontal close to *H. pylori*
Hirsch *et al.*^10^	Original Research	Germany	10	PCR	Periodontal close to *H. pylori*
Salazar *et al.*^37^	Original Research	USA	131	Seropositive	Periodontal close to *H. pylori*
Fernandez *et al.*^7^	Original Research	Mexico	200	Seropositive	Periodontal close to *H. pylori*
Tsami *et al.*^9^	Original Research	Greece	35	Seropositive	Periodontal close to *H. pylori*
Chaudhry *et al.*^25^	Original Research	Pakistan	100	PCR	Periodontal close to *H. pylori*
Chen *et al.*^30^	Original Research	China	173	Saliva HP test	Periodontal close to *H. pylori*
Navabi *et al.*^17^	Review Article	Iran	1861	PCR	Periodontal close to *H. pylori*
Bago *et al.*^39^	Original Research	Croatia	56	PCR	Periodontal close to *H. pylori*
Namiot *et al.*^23^	Original Research	Poland	155	HP antigen	No close relation
Silva *et al.*^24^	Original Research	Brazil	115	PCR	Periodontal close to *H. pylori*
Cellini *et al.*^38^	Original Research	Italy	19	PCR	Periodontal close to *H. pylori*
Dye *et al.*^6^	Original Research	USA	4504	Seropositive	Periodontal close to *H. pylori*
Butt *et al.*^21^	Original Research	Pakistan	178	CLO test	Periodontal close to *H. pylori*
Hardo *et al.*^19^	Original Research	UK	62	PCR	Periodontal close to *H. pylori*

**Table 3:** Studies show the association of oral and stomach *H. pylori* infection

*Author*	*Country*	*No*	*Method*	*Conclusion*
Yee *et al.* ^20^	China	>10 000	HPS	Association of Oral and stomach *H. pylori*
Medina *et al.*^51^	Argentina	8	PCR	Association of Oral and stomach *H. pylori*
Eskandari *et al.*^50^	Iran	67	PCR	
Rasmussen *et al.*^47^	Brazil	78	PCR	Association of Oral and stomach *H. pylori*
Loster *et al.*^55^	Poland	46 dentists	Serological test	Association of Oral and stomach H. pylori
Liu *et al.*^54^	China	443	PCR	Association of Oral and stomach *H. pylori*
Al Asqah *et al.*^53^	Saudi Arab	101	Urease test	Association of Oral and stomach *H. pylori*
Silva *et al.*^58^	Brazil	62	PCR	Association of Oral and stomach *H. pylori*
Zaric *et al.*^49^	Serbia		PCR	Association of Oral and stomach *H. pylori*
Jia *et al.*^67^	China	56	PCR	Association of Oral and stomach *H. pylori*
Morales *et al.*^48^	Mexico	65	PCR	Association of Oral and stomach *H. pylori*
Oshowo *et al.*^41^	UK	208	PCR	Association of Oral and stomach *H. pylori*
Maplstone *et al.*^40^	UK	13	Nested PCR	Association of Oral and stomach *H. pylori*

**Table 4:** Eradication on stomach *H. pylori* infection had no effects on oral *H. pylori*

*Author*	*Country*	*No*	*Time after eradication*	*Positive H. pylori in Oral*	*Positive H. pylori in Stomach*
Adadi *et al.*^32^	Iran	132	After eradication	Patients carrying *H. pylori*	Patients carrying *H. pylori*
Wang *et al.*^1^	China	159	4 weeks	49.44%	38.6%
Song and Li^63^	China	391	4 weeks	33.2%	21.6%
Bago *et al.*^39^	Croatia	56	3 months	0	21.7%
Gao *et al.*^62^	China	96	4 weeks	62.8%	32.4%
Zaric *et al.*^49^	Serbia			52%	23%
Gebara *et al.*^61^	Brazil	30	3 months	60%	10%
Miyabayashi *et al.*^60^	Japan	47	4 weeks and 2 years	69.5%	4.2%
Dore-Davis *et al.*^59^	Swiss	22	4 weeks	57%	

**Table 5:** Articles published since 1989 regarding culture of *H. pylori* in oral cavity

*Author*	*Country*	*N0*	*Dental plaque*	*Saliva*	*Teeth*
Krajden *et al.*^80^	Canada	71	1	0	
Oshowo *et al.*^51^	UK	180	2	0	
Cheng *et al.*^30^	UK	122	0	0	1
Luman *et al.*^82^	UK	109	0	0	0
Allaker *et al.*^83^	UK	100	0	0	0
Bernander *et al.*^84^	Sweden	114	0	0	0
Pustorino *et al.*^85^	Italy	83	5	0	0
Khandaker *et al.*^86^	UK	81	12	0	0
Ishihara *et al.*^87^	Japan	82	0	0	0
Hardo *et al.*^19^	UK	62	0		0
Majmudar *et al.*^88^	India	40	40		
Cellini *et al.*^89^	Italy	31	1		
Wahlfors *et al.*^90^	Finland	29	0	0	
Paronnet *et al.*^42^	USA	26		3	
Namavar *et al.*^91^	Holland	20	1		
D'Alessandro and Seri^92^	Italy	20	16		
Ferguson *et al.*^93^	USA	16		1	
Bickley *et al.*^94^	UK	15		0	
Zhent *et al.*^30^	China	72		31	
Chen *et al.*^30^	China	173		69	
Zheng *et al.*^96^	China	163		42	
Jiang *et al.*^97^	China	50		13	
Xu *et al.*^98^	China	98		21	
Agarwal *et al.*^99^	India	30	9		
Czesnikiewicz-Guzik *et al.*^100^	Poland	100	45	55	
Czesnikiewicz-Guzik *et al.*^101^	Poland	100	46	54	
Loster *et al.*^102^	Poland	46		22	20
Sudhakar *et al.*^103^	India	50	10		
Teoman al.^104^	Turkey	67	17		
Umeda *et al.*^105^	Japan	57	18		
Goosen *et al.*^106^	South Africa	58	2		
Majmudar *et al.*^88^	India	40	40		
D'Alessandro *et al.*^92^	Dell’ Aquila	20	16		
Namavar *et al.*^91^	Netherlands	20		3	
Me'graud *et al.*^122^					
Wang *et al.*^1^	China	159		94	
